# Screening of wheat grains enriched with wall-bound phenolic compounds

**DOI:** 10.1016/j.mex.2023.102245

**Published:** 2023-06-07

**Authors:** Yi-Lin Gong, Jin-Ying Gou

**Affiliations:** a School of Life Sciences, Fudan University, Shanghai, 200438, China; b College of Agronomy and Biotechnology, China Agricultural University, Beijing 100193, China

**Keywords:** *Screening phenolic-enriched grains*, Wheat, Ferulic acid, Ultraviolet spectrophotometry, Liquid chromatography

## Abstract

Phenolic compounds are dominant antioxidant factors in whole grains and are essential quality traits in future breeding programs. We proposed a robust set of methods for extraction, screening, and quantitative analysis of soluble and wall-bound (WB) phenolic compounds from fine powder and fine powder products using a 96 Wells UV Flat Bottom and subsequent UHPLC-DAD validation of candidate samples. The plate-UHPLC strategy significantly simplifies the screening of phenolic-enriched grains, reduces the screening cost, saves harmful organic chemicals, and contributes to developing novel health-promoting varieties.

## Graphical abstract

**Figure:**
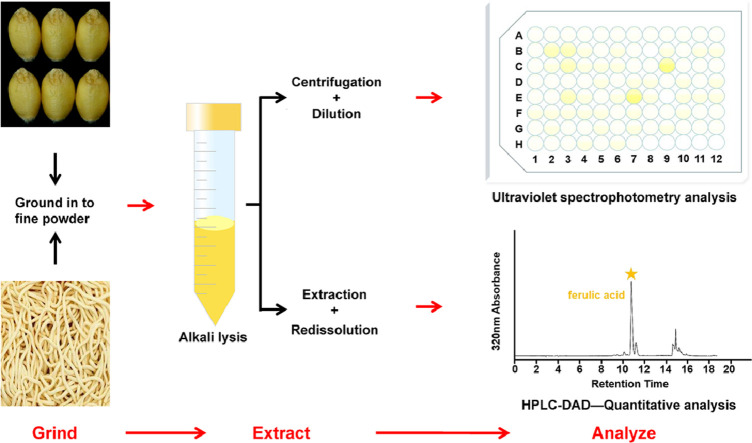

Specifications table

**Table:** 

Subject Area:	Agricultural and Biological Sciences
More specific subject area:	Metabolite analysis
Protocol name:	Efficient screen and quantification of phenolic compounds in wheat grain
Name and reference of original method:	Zhang et al. [1] Food Chem, 385, 132634. Wang et al. [2] Food Chem, 303, 125363.
Reagents/tools:	hydrochloric acid (HCl, analytically pure), sodium hydroxide (NaOH, analytically pure), ferulic acid (FA, 99%), methanol (UPLC grade), acetic acid (analytically pure), and ethyl acetate (analytically pure) from Sigma-Aldrich (St. Louis, MO, USA).
Experimental design:	Grind grains and extract wall-bound phenolics, Extract the phenolics and read OD320 nm in a UV Flat bottom 96-well plate; Select differential ones and confirm by UHPLC.
Trial registration:	Not applicable
Ethics:	Not applicable
Value of the Protocol:	Save 90% of time than UHPLC; Save 90% of chemicals than UHPLC strategy.

**Description of protocol:**

**Methods details**

## Background

The antioxidant characteristic of wholegrain phenolics benefits human health by lowering the risks of cardiovascular disease (CVD), gastrointestinal (GI) cancer, and diabetes [3]. Cell wall-bound (WB) phenolic acids, mainly ferulates (FA), account for over 70% of the total phenolic acid concentration [4]. FA primarily exists in the cell walls of the seed coat and aleuronic layer than in endosperm [5]. Many phenolic compounds have been detected and quantified in plant samples by ultra-high-performance liquid chromatography with a diode array detector (UHPLC-DAD) [6].

We screened wheat mutants enriched with ferulic acid by UHPLC-DAD to increase the antioxidant potential of foods. Screening mutant grains led to identifying mutants accumulating ferulates in the grains [2]. The biochemical and metabolite study revealed that an SNP in the tetraploid wheat increased the FA content and the antioxidant potential, which benefits consumers [1]. Nevertheless, UHPLC experiments take a long time and consume many harmful organic chemicals, which are unfriendly to the environment. To resolve the above limitations, we added a UV absorbance-based plate screening step before the UHPLC validation of candidates to save time and chemicals.

### Chemicals and reagents

We purchased the following chemicals: hydrochloric acid (HCl, analytically pure), sodium hydroxide (NaOH, analytically pure), ferulic acid (FA, 99%), methanol (UPLC grade), acetic acid (analytically pure), and ethyl acetate (analytically pure) from Sigma-Aldrich (St. Louis, MO, USA).

**Step 1: Wheat sample preparation**

We recommend the following method for the rapid preparation of large quantities of trace samples (less than six grains):

1. Cover two grains with foil and crush them with the pliers.
2. Ground the samples into a fine powder (60 meshes) with two stainless steel beads in high-throughput grinding equipment (Wanhon, Shanghai, China).
3. Set the grinding program as 20 HZ for 4 min, 25 HZ for 4 min, 30 HZ for 4 min, 40 HZ for 1 min twice, 50 HZ for 1 min twice, 60 HZ for 1 min twice, 65 HZ for 30 s 20 times.
4. Store the samples at -80°C until subsequent chemical analyses.

**Step 2: Extraction of cell wall-bound (WB) phenolic compounds**

1. Wash the ground samples (100 mg ±0.5) with 1 mL methanol (80% in water).
2. Centrifuge at 13,000g for 10 min; carefully drain out the supernatant
3. Repeat twice to eradicate soluble flavonoids.
4. Resuspend the debris in 4N NaOH solution (3mL).
5. Shake the samples at 200 rpm overnight at 37 °C.
6. Centrifuge the samples at 10,000g for 20 min.
7. Extract 100 µL supernatant for subsequent chemical analyses.

**Step 3: Ultraviolet spectrophotometry analysis of phenolic acid**

1. Mixed 100 µL supernate with 300 µL 4 N NaOH.
2. Remove 200 µL into a UV Flat bottom 96-well plate (Thermo SCIENTIFIC, Waltham, MA, USA).
3. Read the absorbances at 320 nm in a MULTISKAN GO (Thermo SCIENTIFIC, Waltham, MA, USA).
4. Calculate phenolic acid content according to the standard curve with 4N NaOH as the negative control.

### HPLC-DAD—Quantitative analysis of phenolic compounds

**Step 4: Wheat sample preparation and pasta-making**

1. Grind the grains into whole wheat flour (60 meshes) in a stone mill.
2. Mix the whole wheat flour and 25% water (w/w) to prepare raw pasta.
3. Prepare threaded pasta with ATLAS 150 (MARCATO S. r.l., Campodarsego, Italy).
4. Cook the raw pasta for ten minutes in boiled water.
5. Wash the pasta twice with ddH_2_O to remove potential residue soup contamination.
6. Dry the raw and cooked pasta in a 65°C oven to achieve constant weight.
7. Grind the samples into a fine powder in the high-throughput grinding equipment for 60 s at 60 HZ.
8. Store the samples at -80°C until further analyses.

**Step 5: Extraction of wall-bound (WB) phenolic compounds**

1. Add methanol (80% in water) at ten microliters per mg into dried powders.
2. Sonicate the samples in a water bath for 30 min.
3. Centrifugate at 15,000 g for 15 min.
4. Wash the samples with methanol (80% in water) three times to remove the soluble part.
5. Treat the debris with 4N NaOH (3mL) containing p-toluic acid (10 µg, internal standard, IS).
6. Shake the samples overnight at 37 °C.
7. Adjust the samples with 2 mL hydrochloric acid (6N) to pH 4.0.
8. Add 600 µL ethyl acetate (water-saturated) for extraction and mix them well by vigorous shaking.
9. Centrifugated at 10000 g for 15 min,
10. Collect the supernatant and repeat the wash three more times.
11. Pass the samples through 1 mL anhydrous sodium sulfate in a 150 mm Glass Pasteur straw Jiangsu Shitai, Jiangsu, China) to remove any polar contamination.
  Dry the samples under a stream of nitrogen gas.
12. Resolve the samples with HPLC-grade methanol at one microliter per mg dry weight grain powder ratio and analyze by UHPLC [1].

**Step 6: Quantitative phenolic acid analysis**

The analysis was performed using an Agilent 1290 Infinity system with a CNW Athena C18-WP HPLC Column (3um, 4.6 × 250mm) coupled with a DAD detector (Agilent, Santa Clara, CA, USA) [7,8]. 5 µL injection volume was used with a total flow rate of 1 mL/min over a full run time of 18 min. Mobile phase A consisted of HPLC-grade ddH_2_O with 0.1% acetic acid (v/v), while mobile phase C was acetonitrile with 0.1% acetic acid (v/v). The gradient details are in Table 1.

**Table 1:** The regent gradient that was used for the separation and identification of WB phenolic compounds.

Time(min)	Flow rate(mL/min)	%A	%C
0	1	80.0	20.0
5	1	70.0	30.0
10	1	70.0	30.0
12	1	0.0	100.0
13.5	1	0.0	100.0
16	1	40.0	60.0
17	1	80.0	20.0
18	1	80.0	20.0

## Results and discussion

**Fig. 1:**
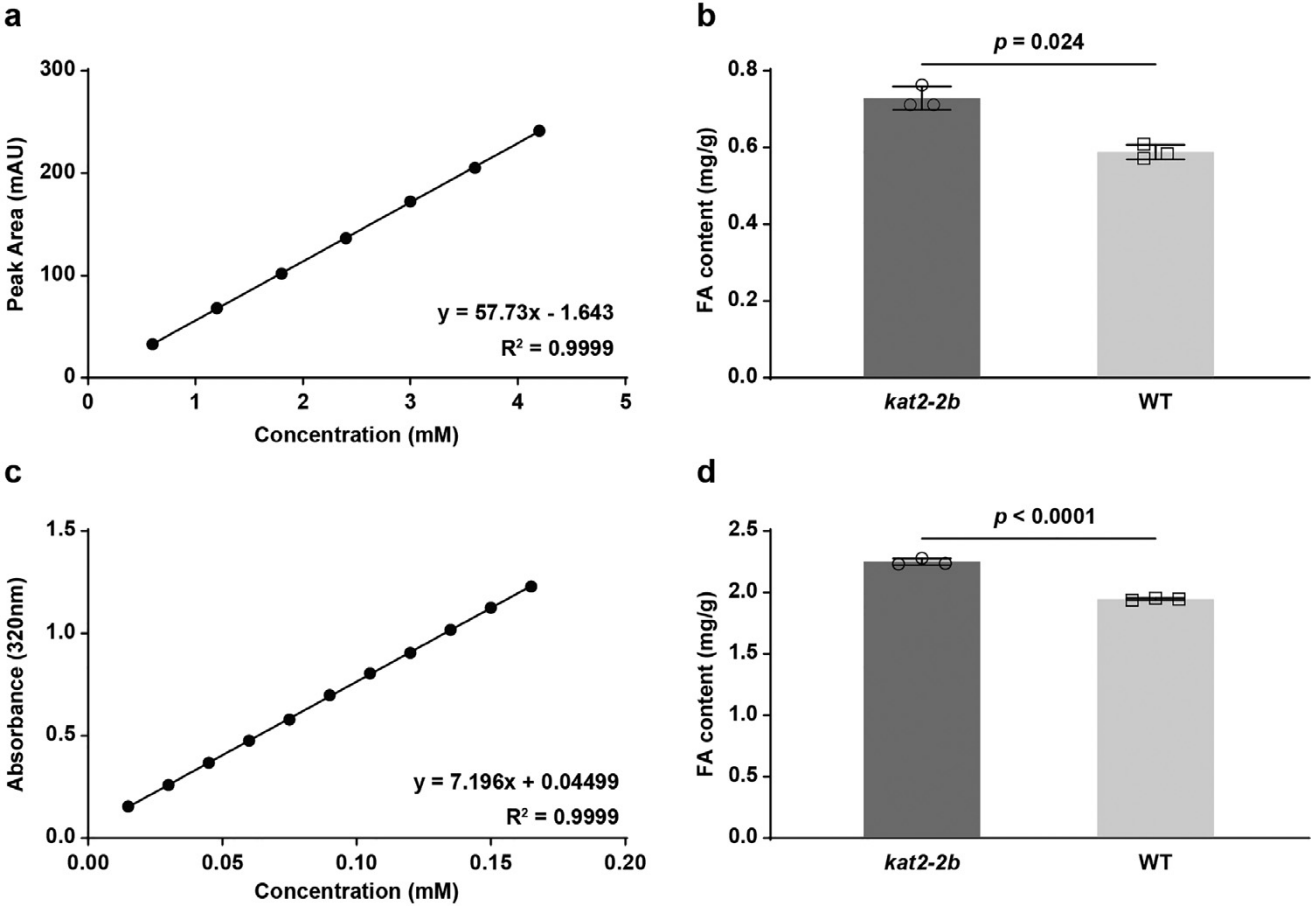
Contents of phenolic acid in the whole wheat flour of the *kat-2b* mutant and WT by UHPLC and ultraviolet spectrophotometry. (a-b) The standard curve (a) and the relative phenolic acid contents (b) in *kat-2b* mutant and WT by the UHPLC. (c-d) The standard curve (c) and the relative phenolic acid contents (d) in *kat-2b* mutant and WT in the ultraviolet spectrophotometry method. Data represented mean ± SD. The two-tailed unpaired Student’*s t*-test indicates *p*-values. *N* = 3.

Our data demonstrated that the UV-spectrophotometer screening method effectively screened phenolic-enriched grains, although its precision and sensitivity were weaker than UHPLC. Given the cost and time reductions, the UV spectrophotometer/UHPLC strategy improves efficiency. Analyzing 96 samples take less than 15 min compared with 1920 min by the UHPLC method [1]. If the top ten samples were selected and analyzed in detail, it would save 90% of chemicals by the UV spectrophotometer/UHPLC strategy. Therefore, the above scenario represents a time-saving, cost-effective, and environmentally friendly method for future health-promoting grain breeding programs (Fig. 1).

## Funding

This work was supported by the National Natural Science Foundation of China (31972350).

## CRediT authorship contribution statement

**Yi-Lin Gong:** Methodology, Data curation, Writing – original draft. **Jin-Ying Gou:** Conceptualization, Writing – review & editing.

## Declaration of Competing Interest

The authors declare that they have no known competing financial interests or personal relationships that could have appeared to influence the work reported in this paper.
